# Serologic Evidence of Highly Pathogenic Avian Influenza A(H5N1) Virus Infection in a Veterinary Professional Exposed to an Infected Domestic Cat — Los Angeles County, California, December 2024–January 2025

**DOI:** 10.15585/mmwr.mm7517a1

**Published:** 2026-05-07

**Authors:** Aisling Vaughan, Allison Joyce, Elizabeth Traub, Mellissa Jae, Emily Beeler, Erick Paiva, Kristopher Ananian, Crystal Holiday, Stacie Jefferson, Jessica Richardson, Cortney Munna, Cynthia Chan, Tamerin Scott, Noah Kojima, Tanya Seneviratne, Alexandra Mellis, Sonja J. Olsen, Nicole Green, Matt Feaster, Dawn Terashita, Sharon Balter, Min Z. Levine, Jamie Middleton, Annabelle de St. Maurice

**Affiliations:** ^1^Epidemic Intelligence Service, CDC; ^2^Acute Communicable Disease Control, Los Angeles County Department of Public Health, Los Angeles County, California; ^3^Veterinary Public Health, Los Angeles County Department of Public Health, Los Angeles County, California; ^4^Influenza Division, National Center for Immunization and Respiratory Diseases, CDC; ^5^Public Health Laboratory, Los Angeles County Department of Public Health, Los Angeles County, California; ^6^Pasadena Department of Public Health, Los Angeles County, California.

**Summary**

**What is already known about this topic?**

Transmission of influenza A(H5N1) viruses from domestic cats to humans has not been documented.

**What is added by this report?**

During November 2024–January 2025, a total of 139 persons exposed to 19 A(H5N1)-infected domestic cats that consumed raw animal products were identified in Los Angeles County, California. Among 25 exposed persons who received serologic testing, one asymptomatic veterinary professional had serologic evidence of A(H5N1) infection after occupational exposure to an A(H5N1)-infected cat.

**What are the implications for public health practice?**

These findings provide evidence of zoonotic transmission of influenza A(H5N1) virus from domestic cats to humans. Pet owners are advised not to feed raw animal products to cats. Veterinary professionals should be aware of infection risks, use appropriate personal protective equipment, and adhere to recommended infection control practices to reduce the risk for zoonotic transmission of influenza A(H5N1).

## Abstract

Since 2021, avian influenza A(H5N1) clade 2.3.4.4b viruses have spread widely among wild birds and domesticated poultry in the United States, with sporadic spillover into mammals. During November 2024–January 2025, 19 domestic cats in Los Angeles County, California, became ill after consumption of commercially purchased raw milk, raw meat, or raw pet food; nine cats tested positive for influenza A(H5N1) virus (clade 2.3.4.4b genotype B3.13). Overall, 139 persons were exposed to the 19 infected cats, and all were monitored for symptoms. Although 30 persons reported influenza-like illness symptoms, none received a positive influenza A(H5) reverse transcription–polymerase chain reaction (RT-PCR) test result. In April 2025, the Los Angeles County Department of Public Health and CDC invited all exposed persons to participate in an influenza A(H5N1) serosurvey to determine whether transmission of influenza A(H5N1) virus occurred, including in those without symptoms. Sera from 25 (18%) of the 139 exposed persons were tested. Among these, antibodies specific to A(H5N1) clade 2.3.4.4.b (antigenically similar to the clade 2.3.4.4.b influenza A[H5N1] virus isolated from the infected cats) were detected in serum from one veterinary professional, who was asymptomatic. This person did not use respiratory or eye protection during the exposure, did not report influenza-like illness after the exposure, and reported no other known risk factors for A(H5N1) infection. These findings represent serologic evidence of possible transmission of influenza A(H5N1) clade 2.3.4.4.b virus from a domestic cat to a human, highlighting concerns about potential cat-to-human transmission of influenza A(H5N1) virus and the importance of infection control practices in veterinary settings.

## Introduction

### Influenza A(H5N1) Infections in the United States

Since 2021, highly pathogenic avian influenza A(H5N1) clade 2.3.4.4.b viruses have spread widely among wild birds and domesticated poultry in the United States, with increasing spillover into mammals, including dairy cattle, domestic cats, and humans (*1*,*2*). A majority of human cases in the United States have been mild and associated with known exposure to ill or infected animals (*1*). Influenza A(H5N1) infections in cats are often associated with severe disease and have been linked to consumption of unpasteurized (raw) milk, raw meat, or raw pet food or exposure to dairy farm environments (*3*,*4*). To date, domestic cat-to-human transmission of influenza A(H5N1) has not been documented; however, close contact between cats and humans could create opportunities for exposure, particularly among pet owners and veterinary personnel.

### Diagnosis of Influenza A(H5N1) Infection in Symptomatic Cats in Los Angeles County, California

During November 2024–January 2025, the Los Angeles County Department of Public Health (LACDPH) received reports of 19 domestic cats from five households in Los Angeles County, California that became ill with severe respiratory, hepatic, or neurologic signs. Fourteen died or were euthanized. LACDPH reviewed veterinary records, and the cat owners were interviewed. Nine cats were tested; all specimens tested positive for influenza A(H5N1) virus; sequencing analysis confirmed clade 2.3.4.4b genotype B3.13 influenza A(H5N1) virus.

All of the cat owners reported feeding their pets commercially purchased raw milk, raw poultry, or raw pet food in the weeks preceding illness onset; some of the products had tested positive for influenza A(H5N1). Among all 19 cats, 14 (74%) had been evaluated at 10 different veterinary practices. All suspected or confirmed feline cases of influenza A(H5N1) infection^†^ were detected through veterinarian reports, commercial laboratory reports, routine influenza A reverse transcription–polymerase chain reaction (RT-PCR) testing of brain tissue from euthanized cats submitted to the LACDPH laboratory for rabies testing, and cats that were epidemiologically linked to a confirmed influenza A(H5N1) infected feline case.

The risk for transmission of influenza A(H5N1) infection from cats to humans is unknown. To guide public health action, LACDPH conducted a public health investigation to assess whether influenza A(H5N1) infection occurred among pet owners or veterinary professionals who were exposed to the infected cats.

## Methods

### Investigation of Contacts

LACDPH interviewed pet owners in the five households with affected cats, and reviewed lists of staff members provided by managers at the 10 veterinary clinics where 14 of the cats had been evaluated and at an animal control agency involved in transportation of cat carcasses to LACDPH’s laboratory for testing. A total of 139 persons with potential exposure to domestic cats with suspected or confirmed influenza A(H5N1) virus infection were identified. Standardized interviews were conducted with symptomatic persons to ascertain their exposure history as well as any signs or symptoms of illness. Exposure was defined as having handled or participated (within 6 ft [1.8 m]) in the clinical care of an animal with suspected or confirmed influenza A(H5N1) infection, regardless of whether personal protective equipment (PPE) had been used. Identified exposed persons were enrolled in active symptom monitoring for 10 days after their last known exposure and offered RT-PCR testing of a nasopharyngeal swab for influenza A(H5N1). Nasopharyngeal specimens collected from exposed persons were tested at LACDPH’s laboratory using the multiplex RT-PCR BioFire Respiratory Panel FilmArray (BioFire Diagnostics).^§^ Unsubtypeable influenza A–positive specimens underwent reflex testing using the CDC Human Influenza Virus Real-Time RT-PCR, Influenza A/H5 Subtyping Kit (*5*). The antiviral oseltamivir was offered to exposed persons on a case-by-case basis if symptoms were clinically compatible with influenza A(H5N1) infection and <5 days had elapsed since the last exposure.

### Serologic Investigation

In April 2025, the 139 persons who had potentially been exposed since December 2024 were invited to participate in a serologic investigation to identify evidence of influenza A(H5N1) infection. Participants were asked to complete a standardized questionnaire that captured demographic information; recent respiratory illness history; detailed exposure history, including exposure to raw animal products, wild birds, poultry or cattle; PPE use; and information on other potential risk factors for influenza A(H5N1) infection. A single venous blood specimen was collected from each serosurvey participant. Serologic analyses were performed at CDC in biosafety level 3 enhanced laboratories, following previously described established protocols (*6*). All serum samples were tested for evidence of recent infection by microneutralization (MN) and hemagglutination inhibition (HI) assays using two wild-type influenza A(H5N1) viruses: 1) A(H5N1) clade 2.3.4.4b B3.13 (A/Michigan/90/2024) and 2) A(H5N1) clade 2.3.4.4b D1.1 (A/Washington/240/2024). Samples that tested positive underwent serum adsorption using hemagglutinin head domain from influenza A(H1N1)pdm09 virus (A/Wisconsin/588/2019) and A(H3N2) (A/Darwin/6/2021) to eliminate cross-reactivity with seasonal influenza viruses. Samples with a geometric mean titer (GMT) ≥40 in both MN and HI assays were considered seropositive for influenza A(H5N1). This activity was reviewed by LACDPH and CDC, deemed not research, and conducted consistent with applicable federal law and CDC policy.^¶^

## Results

### Identification and Monitoring of Exposed Persons

The 139 persons who had contact with a cat with suspected or laboratory-confirmed influenza A(H5N1) infection included 11 from five households, 126 from 10 veterinary practices, one from an animal control agency, and one from a local health department (Figure). All exposed persons were monitored for symptoms for 10 days after exposure and offered testing; 33 (24%) persons agreed to receive testing. Daily symptom monitoring was conducted using Research Electronic Data Capture (REDCap; Vanderbilt University): participants received a daily automated text message prompt that linked to a brief questionnaire. Overall, 30 (22%) persons reported influenza-like illness symptoms including runny nose, cough, sore throat, fatigue, muscle aches, headache, and sneezing. The average interval from exposure to symptom onset was 7 days. One person reported symptoms on two occasions, after exposures to two different cats, 2 weeks apart.

**FIGURE:**
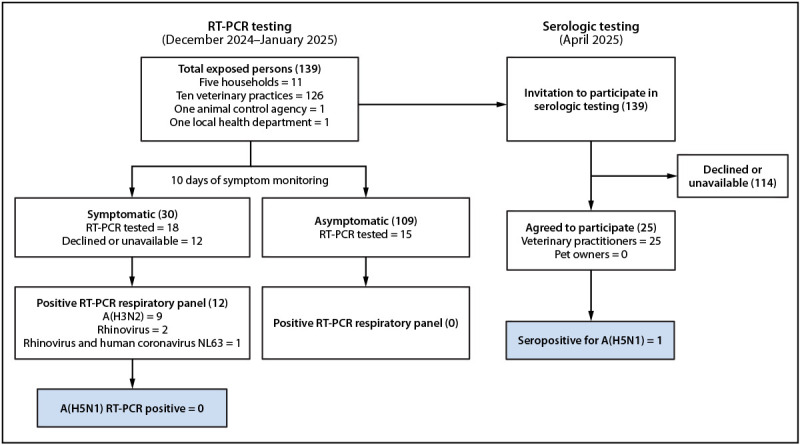
Reverse transcription–polymerase chain reaction* and serologic testing results for persons exposed to domestic cats with suspected or confirmed influenza A(H5N1) infection — Los Angeles County, California, December 2024–January 2025 **Abbreviation: **RT-PCR = reverse transcription–polymerase chain reaction. * Among symptomatic persons, 18 of 30 agreed to RT-PCR testing. Among asymptomatic persons, the 15 persons who received RT-PCR testing included 14 asymptomatic veterinary professionals and one asymptomatic pet owner who agreed to testing.

### Detection of Respiratory Viruses in Nasopharyngeal Swab Specimens

During December 2024–January 2025, nasopharyngeal specimens were collected from 33 persons, including 18 symptomatic veterinary staff members who agreed to testing after development of symptoms, 14 asymptomatic veterinary professionals, and one asymptomatic pet owner. The median interval between the most recent exposure date and specimen collection was 8 days (range = 1–13 days). Specimens from 19 (58%) persons who received testing were collected >7 days after the last exposure. No specimen tested positive for influenza A(H5N1). Among 12 (36%) respiratory panel RT-PCR–positive test results, nine were positive for seasonal influenza A(H3N2), two for rhinovirus, and one for both rhinovirus and an endemic coronavirus (coronavirus NL63). No asymptomatic person received a positive test result for any virus on the respiratory panel.

### Detection of Antibodies to Influenza A(H5N1) in an Exposed Person

Among all 139 exposed persons, 25 (18%) agreed to participate in the influenza A(H5N1) serosurvey. The average interval between exposure and serum collection was 104 days (range = 35–137 days). Among all 25 serum samples, one sample (4%), which was collected from an asymptomatic veterinary professional 120 days after exposure to an ill cat, tested positive for both neutralizing and HI antibodies (GMT ≥40) against both clade 2.3.4.4.b B3.13 (MN = 60; HI = 40) and D1.1 (MN = 113; HI = 80) influenza A(H5N1) viruses. These viruses are antigenically similar to the clade 2.3.4.4b influenza A(H5N1) virus isolated from the infected cats.

### Retrospective Investigation of the Seropositive Veterinary Professional

**Cat’s illness and test results.** In early December 2024, the seropositive veterinary professional was exposed to an ill indoor housecat that was ultimately evaluated over a period of 11 days at four veterinary practices for upper respiratory signs, radiographic evidence of pulmonary lesions, transient ataxia and bilateral hind limb weakness, and progressive bilateral uveitis with retinal hemorrhage and detachment and blindness. All clinical signs occurred after the cat ate a commercial raw pet food (poultry blend). The cat survived but with permanent vision impairments. During these veterinary clinic visits, 31 persons were exposed to the cat.** The cat’s clinical evaluation included physical and ophthalmologic examinations, blood collections, thoracic radiographs, an echocardiogram, and an abdominal ultrasound. At the cat’s second veterinary clinic visit, deep pharyngeal and conjunctival specimens were collected and pooled, and a commercial veterinary laboratory conducted testing for several pathogens (including influenza A virus) using a feline respiratory RT-PCR panel; 7 days later, results were reported to the Veterinary Public Health program (VPH) at LACDPH as positive for both feline calicivirus and influenza A (unsubtypeable). VPH arranged for the total nucleic acids from the specimen to be forwarded to the U.S. Department of Agriculture National Veterinary Services laboratory. Whole genome sequencing identified the virus as influenza A(H5N1) clade 2.3.4.4b B3.13 (A/cat/California/038279-001/2024; GISAID isolate ID: EPI_ISL_19645032) 2 weeks later.

Interviews with the cat owner and veterinary staff members revealed that the cat had received care at four practices during the week preceding the release of the positive result (unsubtypeable influenza A) from the RT-PCR feline respiratory panel, which contributed to a lack of awareness of the veterinary staff member’s exposure risk. Moreover, after the initial positive result became available, the public health significance was not conveyed to the pet owner or other veterinary staff members until they were contacted by LACDPH. As a result, staff members were unaware of the risk for zoonotic influenza A infection, including at the clinic where the veterinary professional was exposed.

**Assessment of the asymptomatic veterinary professional.** The veterinary professional remained asymptomatic after the exposure and had received a negative influenza A virus test result 7 days after exposure to the cat. Because this person was asymptomatic, no interview was conducted at the time of exposure, and information regarding the exact nature of the interaction between the veterinary professional and the infected cat was not available. More detailed risk factor information collected during the serosurvey indicated that this person routinely engaged in multiple clinical duties, including restraining animals; assisting with veterinary surgery; administration of inhalation anesthesia; performing cardiopulmonary resuscitation; collection of nasopharyngeal, blood, fecal, rectal, saliva, and urine samples; performing endotracheal intubation or other airway procedures; and cleaning examination rooms. Staff members at this facility were reported to routinely wear gloves but no other PPE during examinations. The veterinary professional had no known exposure to another cat with suspected or confirmed H5N1 infection, and reported no exposure to backyard poultry, wild birds, or dairy cattle; no consumption of raw animal products; and no underlying medical conditions. This person had not received either the 2024–25 seasonal influenza vaccine (which is not intended to prevent influenza A[H5N1] infection) or postexposure antiviral prophylaxis.

## Discussion

This investigation identified serologic evidence of influenza A(H5N1) infection in an asymptomatic veterinary professional who was occupationally exposed to a domestic cat with confirmed influenza A(H5N1) virus infection; the person received a negative RT-PCR influenza A(H5N1) virus test result 1 week after exposure. These findings provide serologic documentation of possible transmission of avian influenza A(H5N1) clade 2.3.4.4.b virus from a domestic cat to a human. Serologic testing identified evidence of a likely infection that would otherwise have remained undetected, contributes to the understanding of A(H5N1) transmission from animals to humans, and highlights the potential use of serologic testing in identifying infections.

Zoonotic transmission involving domestic cats and other felids has been previously reported, including low pathogenicity avian influenza A(H7N2) virus infection in a symptomatic veterinarian who was exposed to infected cats at an animal shelter in New York City (*7*) and seroconversion among zoo workers after exposure to captive tigers during an influenza A(H5N1) outbreak in Thailand (*8*).

Although the precise nature of exposure in the veterinary clinic is not known, this finding raises concern about zoonotic transmission of influenza A(H5N1) virus from domestic cats to humans and reinforces the need for heightened awareness among pet owners and veterinary professionals, as well as strict infection control practices in veterinary settings (*9*). The occurrence of this exposure during a seasonal influenza A(H3N2) virus outbreak highlights the risk for co-infection and potential for reassortment between seasonal and avian influenza viruses, which could lead to the emergence of a novel viral strain capable of sustained human-to-human transmission and pandemic potential.

### Limitations

The findings in this report are subject to at least two limitations. First, RT-PCR testing and serologic testing were not performed for all persons; therefore, some infections might have been missed. Second, serologic testing was performed 4–5 months after exposure, at which time antibody responses might have waned. Collection of acute and convalescent serology specimens was not feasible in this investigation; however, this step should be considered during future influenza A(H5N1) virus outbreaks in animals.

### Implications for Public Health Practice

Detection of influenza A(H5N1) in domestic cats and transmission to a veterinary professional highlight a source of potential human exposure. Given the close contact that is common between cats and humans, continued vigilance is warranted. The cats described in this report were all reported to have consumed raw meat, raw pet food, or raw milk, products that have been documented to be sources of H5N1 infection in pets (*10*). Feeding these products to pets could increase their risk for infection with influenza viruses. Animal health alerts disseminated by LACDPH warned of severe illness and death in cats associated with consumption of influenza A(H5N1)-infected raw dairy, raw meat, and raw poultry or raw pet food diets. Pet owners are advised not to feed cats raw milk or other raw animal products. Veterinarians should consider influenza A(H5N1) in cats with acute respiratory or neurologic illness and follow appropriate infection prevention practices, including using PPE, to reduce exposure risk (*9*). Timely detection and a One Health response to influenza A(H5N1) infection in domestic cats, including confirmation, source identification, and prompt evaluation and testing of exposed persons, are essential to reducing additional transmission and the risk for an influenza A(H5N1) pandemic.

## Footnotes

* These authors contributed equally to this report.

† A suspected feline case was defined as an occurrence of illness in a domestic cat with clinical signs compatible with influenza A(H5N1) infection (e.g., fever, lethargy, and inappetence, and possible respiratory illness symptoms such as coughing, sneezing, nasal or ocular discharge, or dyspnea), with rapid progression to neurologic symptoms (e.g., ataxia, blindness, tremors, and seizures) and in some cases, acute death. Suspected cases also had an epidemiologic link to a cat with confirmed infection or to a product with confirmed or suspected contamination (e.g., raw meat, raw pet food, or raw milk). The case met the definition even if testing results of an oral or rectal swab specimen were negative for influenza A by RT-PCR or no RT-PCR test had been performed. Negative RT-PCR results from oral or rectal swabs were not considered sufficient to rule out infection. If RT-PCR testing of brain tissue was performed and the result was negative, the domestic cat was considered negative for influenza A(H5N1) infection. A confirmed feline case was defined as an occurrence of illness in a domestic cat with clinical signs compatible with influenza A(H5N1) infection and a positive H5 RT-PCR result from testing performed at the U.S. Department of Agriculture National Veterinary Services Laboratory.

§ This assay detects multiple respiratory pathogens including influenza A virus subtypes A(H1), A(H3), and A(H1N1)pdm09; influenza B; and other common respiratory viruses.

¶ 45 C.F.R. part 46.102(l)(2), 21 C.F.R. part 56; 42 U.S.C. Sect. 241(d); 5 U.S.C. Sect. 552a; 44 U.S.C. Sect. 3501 et seq.

** In addition to the seropositive person, specimens from 12 exposed persons were tested by RT-PCR; none tested positive for influenza A(H5N1). Three veterinary professionals agreed to participate in the serosurvey; all were asymptomatic and all received negative test results for antibodies against influenza A(H5N1).

## References

[1] CDC. Avian influenza (bird flu). A(H5) bird flu: current situation. Atlanta, GA: US Department of Health and Human Services, CDC; 2025. https://www.cdc.gov/bird-flu/situation-summary/index.html

[2] US Department of Agriculture, Animal and Plant Health Inspection Service. H5N1 influenza. Washington, DC: US Department of Agriculture, Animal and Plant Health Inspection Service; 2026. https://www.aphis.usda.gov/h5n1-hpai

[3] Bonilla-Aldana DK, Bonilla-Aldana JL, Acosta-España JD, Rodriguez-Morales AJ. Highly pathogenic avian influenza H5N1 in cats (*Felis catus*): a systematic review and meta-analysis. Animals (Basel) 2025;15:1441. 10.3390/ani15101441 40427317 PMC12108504

[4] Coleman KK, Bemis IG. Avian influenza virus infections in felines: a systematic review of two decades of literature. Open Forum Infect Dis 2025;12:ofaf261. 10.1093/ofid/ofaf261 40390703 PMC12086332

[5] CDC. Avian influenza (bird flu). Public health and clinical labs: novel influenza A virus testing. Atlanta, GA: US Department of Health and Human Services, CDC; 2025. https://www.cdc.gov/bird-flu/php/severe-potential/novel-influenza-a-virus-testing.html

[6] Levine MZ, Liu F, Bagdasarian N, Neutralizing antibody response to influenza A(H5N1) virus in dairy farm workers, Michigan, USA. Emerg Infect Dis 2025;31:876–8. 10.3201/eid3104.250007 40053378 PMC11950262

[7] Lee CT, Slavinski S, Schiff C, Influenza A(H7N2) Response Team. Outbreak of influenza A(H7N2) among cats in an animal shelter with cat-to-human transmission—New York City, 2016. Clin Infect Dis 2017;65:1927–9. 10.1093/cid/cix668 29020187

[8] Thanawongnuwech R, Amonsin A, Tantilertcharoen R, Probable tiger-to-tiger transmission of avian influenza H5N1. Emerg Infect Dis 2005;11:699–701. 10.3201/eid1105.050007 15890122 PMC3320363

[9] CDC. Avian influenza (bird flu). Managing cats and captive wild animals exposed to bird flu (H5N1). Atlanta, GA: US Department of Health and Human Services, CDC; 2025. https://www.cdc.gov/bird-flu/hcp/animals/index.html

[10] Dhakal J, Bhat S, James J, Otwey RY, Chapagain S, Singh P. Highly pathogenic avian influenza (HPAI) H5N1 in raw pet foods and milk: a growing threat to both companion animals and human health, and potential raw pet food industry liability. J Food Prot 2025;88:100628. 10.1016/j.jfp.2025.100628 41016509

